# Composted versus Raw Olive Mill Waste as Substrates for the Production of Medicinal Mushrooms: An Assessment of Selected Cultivation and Quality Parameters

**DOI:** 10.1155/2013/546830

**Published:** 2013-08-21

**Authors:** Georgios I. Zervakis, Georgios Koutrotsios, Panagiotis Katsaris

**Affiliations:** ^1^Agricultural University of Athens, Laboratory of General and Agricultural Microbiology, Iera Odos 75, 11855 Athens, Greece; ^2^Hellenic Agricultural Organization-Demeter, Institute of Kalamata, Lakonikis 85, 24100 Kalamata, Greece

## Abstract

Two-phase olive mill waste (TPOMW, “alperujo”) is a highly biotoxic sludge-like effluent of the olive-oil milling process with a huge seasonal production. One of the treatment approaches that has so far received little attention is the use of TPOMW as substrate for the cultivation of edible mushrooms. Fifteen fungal strains belonging to five species (Basidiomycota), that is, *Agrocybe cylindracea*, *Pleurotus cystidiosus*, *P. eryngii*, *P. ostreatus*, and *P. pulmonarius*, were evaluated for their efficacy to colonize media composed of TPOMW, which was used either raw or composted in mixtures with wheat straw in various ratios. Qualified strains exhibited high values of biological efficiency (e.g., 120–135% for *Pleurotus* spp. and 125% for *A. cylindracea*) and productivity in subsequent cultivation experiments on substrates supplemented with 20–40% composted TPOMW or 20% raw TPOMW. Only when supplementation exceeded 60% for raw TPOMW, a negative impact was noted on mushroom yields which could be attributed to the effluent's toxicity (otherwise alleviated in the respective composted TPOMW medium). Earliness and mushroom size as well as quality parameters such as total phenolic content and antioxidant activity did not demonstrate significant differences versus the control wheat-straw substrate. The substrates hemicellulose content was negatively correlated with mycelium growth rates and yields and positively with earliness; in addition, cellulose: lignin ratio presented a positive correlation with mycelium growth and mushroom weight for *A. cylindracea* and with earliness for all species examined. TPOMW-based media revealed a great potential for the substitution of traditional cultivation substrates by valorizing environmentally hazardous agricultural waste.

## 1. Introduction

The disposal of wastes and by-products generated by the olive-oil industry are linked with major environmental repercussions because of their high organic content, composition, and seasonality of production. For reducing the huge volumes of olive mill wastewater produced by the widespread three-phase centrifugal systems, two-phase decanters were introduced. In this type of olive mills, the malaxed olive paste is separated from the oily phase with the addition of limited quantities of water. Hence, the only by-product of the extraction process is a sludge-like pomace, known as two-phase olive mill waste (TPOMW or “alperujo”). Nevertheless, TPOMW's properties, that is, moisture content of 60–65%, acidic pH, high content of lipids, and polyphenolics, still pose serious problems related with its effective management and safe disposal [1, 2].

 Several studies dealt with TPOMW detoxification through the development of the appropriate solid-state fermentation methodologies and the addition of suitable co-composting materials for coping with the particular structure of this effluent [3, 4]. Pertinent results and conclusions were further expedited by the evaluation of the final products (TPOMW-based composts) as organic amendments in the cultivation of various crops and for the suppression of soil-borne plant pathogens [5–8]. Apart from the exploitation of mixed microbial communities present throughout the composting process, the potent lignin-degrading enzymatic system of selected wood-rot basidiomycetes was also used to decrease TPOMW phenolics and phytotoxicity [9–11].

 Despite the fact that degradation of olive mill effluents was exhibited in the past by various species of edible ligninolytic macrofungi [12–14], a rather limited amount of information is available on the evaluation of wastes and by-products of the olive-oil industry as substrates for mushroom production. Previous studies focused mainly on the use of olive mill wastewater for *Pleurotus* species cultivation either as the sole ingredient [15], as wetting agent of conventional wheat straw media [16, 17], or even as the main substrate constituent together with extracted olive-press cake [18]. Similarly, Altieri et al. [19] used olive mill by-products from three-phase systems for commercial-scale cultivation of the button mushroom *Agaricus bisporus*. On the other hand, pertinent exploitation of TPOMW has only recently attracted research interest [20, 21] demonstrating that this approach could provide promising alternatives for the valorization of this notorious effluent, especially if the ever growing trend of employing two-phase decanters in Mediterranean countries is taken into consideration.

*Pleurotus ostreatus*, *P. eryngii*, *P. pulmonarius,* and *P. cystidiosus* are among the most commonly occurring *Pleurotus *species and they all present a wide geographical distribution in most temperate regions of the world [22, 23]. Their cultivation is very popular since they can be produced on a large variety of plant residues and agroindustrial by-products [24–27]. On the other hand, *Agrocybe cylindracea* is also a widely distributed wood-rotting fungus [28] reported to grow on a wide range of lignocellulosic substrates [25, 29], and it is among the best candidates for diversifying commercial mushroom markets. In addition, all mentioned edible mushroom species have drawn research attention due to their content in bioactive compounds including antioxidants, vitamins, *β*-glucans, and lectins presenting immunostimulating and antitumor activities [30–33]. However, an important prerequisite for further development of their cultivation is the increase in productivity through the exploitation of novel cheap substrates in order to support a financially viable agroindustrial activity.

The aim of this study was to examine the use of several TPOMW-based media as substrates for the cultivation of five choice edible mushroom species through the evaluation of the appropriate production and quality parameters. In addition, it was investigated whether TPOMW subjected to a composting pretreatment process could further enhance mushroom productivity without any negative impact on quality aspects.

## 2. Materials and Methods

### 2.1. Organisms

Fifteen fungal strains (assigned in five species of the phylum Basidiomycota) were examined in the frame of the present study:

1. *Agrocybe cylindracea*, strains IK10 (Greece), IK21 (Greece), and SIEF0834 (China);
2. *Pleurotus cystidiosus*, strains LGAM P50 (Greece), LGAM P100 (Greece), and D415 (USA);
3. *P*. *eryngii*, strains LGAM63 (Greece), LGAM101 (Greece), and UPA10 (Italy);
4. *P*. *ostreatus*, strains LGAM60 (Greece), LGAM106 (Greece), and LGM850402 (Hungary);
5. *P*. *pulmonarius*, strains LGAM10 (Greece), LGAM26 (Greece), and LGM850403 (France).

All strains tested were isolated from the wild, they were routinely maintained on potato dextrose agar (PDA, Difco), and they are preserved in the fungal Culture Collection of the Laboratory of General and Agricultural Microbiology (Agricultural University of Athens, Greece).

### 2.2. Preparation of TPOMW-Based Media and Assessment of Mycelium Growth Rates

Two-phase olive mill waste (TPOMW, deriving from the respective olive-oil extraction process) was obtained from an olive mill located in the city of Kalamata (Peloponnese, southwest Greece), and it was provisionally stored at 4°C. Apart from the use of raw TPOMW, TPOMW which was previously subjected to a controlled aerobic thermophilic process (composting) was also evaluated. Before composting, olive leaves were added to raw TPOMW as a bulking agent (for improving porosity and facilitating aeration) at a 10% w/w ratio. Composting was performed by means of a static pile placed within a wooden container (0.16 m^3^ volume). Aeration was passive from bottom to top of the pile, and a 2 cm thick layer of polyurethane insulation was used to cover perimetrically the container for minimizing temperature losses. The moisture within the pile was kept at 55–65% of its water-holding capacity by adding water at regular intervals throughout the composting process. Turnings were performed at the end of each thermophilic phase by manually emptying and refilling the container. Temperature and pH values were monitored during the entire process by probes positioned randomly within the pile. Composting was performed for a period of ca. 60 days followed by a 30 days curing period; the active composting phase was considered terminated when the temperature of the pile was close to ambient and (despite turning) further reheating did not occur.

 For determining fungal strains efficiency to colonize substrates, three different media were prepared as follows: raw TPOMW and wheat straw (WS) were mixed in w/w ratios of 60 : 40, 40 : 60, and 20 : 80. In addition, three other substrates were prepared by mixing WS with composted TPOMW (in the same w/w ratios as in the previous case), while a WS-based substrate (without adding TPOMW) was used as control.

 Measurements of linear growth rates were performed to test the ability of these substrates to support growth of the mushroom strains under study. For this purpose, glass “race” tubes (200 × 30 mm) were used as described by Zervakis et al. [34]; three replicates per strain and substrate were inoculated with one agar-plug, taken from the actively growing periphery of a fungal colony developing on PDA (Difco). Incubation of cultures was carried out at 26°C. Mycelium linear growth was recorded daily as previously described [34].

### 2.3. Preparation of Spawn and Mushroom Cultivation Substrates

For inoculation of mushroom cultivation substrates, grain spawn for four selected (on the basis of their growth rates as evidenced in the previous experiment) strains representing *A. cylindracea*, *P. eryngii*, *P. ostreatus,* and *P. pulmonarius* was prepared as described by Philippoussis et al. [25].

 The comparative assessment of fungal strains in the “race” tubes experiment led also to the qualification of seven cultivation substrates for further evaluation: raw and (independently) composted TPOMW in 60 : 40, 40 : 60, and 20 : 80 (w/w) mixtures with WS, plus a plain WS-based substrate serving as control. In each substrate, calcium carbonate was added (2% w/w), pH and moisture content were adjusted to 6.1 and 60–65%, respectively, and it was then supplemented by 5% wheat bran (w/w, in terms of dry weight). Three replicates per substrate and strain were used. Polypropylene-autoclavable bags were filled with substrate and were sterilized for one hour at 1.1 atm. Spawn inoculation was carried out along the central vertical axis of the bag, at a rate of 3% (w/w), by using sterile hollow copper tubes. Colonization of the substrates and fructification took place in growth chambers as previously described [25]. In brief, incubation of the cultures within the tightly closed bags took place at 25°C in the dark; when substrates were fully colonized, relative air humidity was set at 95 ± 2%, temperature was decreased by 7°C, and illumination was provided (700 lux m^−2^, 12 h day^−1^ with fluorescent lamps). After primordium formation, CO_2_ levels were maintained at less than 1200 ppm, relative humidity at 80 ± 2%, and illumination at 1000 lux m^−2^ (12 h day^−1^ with fluorescent lamps).

### 2.4. Crop Yield Parameters

Three mushroom production flushes for *P. ostreatus* and *P. pulmonarius* and two flushes for *P. eryngii* and *A. cylindracea* were collected during the cropping period. Mature basidiomata were harvested at the same time each day, counted and weighted. Among the parameters evaluated to test the suitability of the substrates under study for the cultivation of the four mushroom species were (a) earliness, defined as the time elapsed between the day of inoculation and the day of primordia formation, (b) yield, expressed as fresh weight of mushrooms harvested, (c) biological efficiency (BE), calculated as the percentage ratio of fresh mushrooms weight over the dry weight of the substrate, (d) productivity, expressed as the ratio of yield over the number of cultivation days, and (e) average mushroom weight, measured as the ratio of the total mushroom yield over the number of individual basidiomata produced.

### 2.5. Chemical Analyses and Phytotoxicity Tests

Measurements of pH were performed with a Scott Geräte TR156 pH-meter by using a 1 : 2 sample to water ratio. Total nitrogen of the substrates used was determined by wet digestion of finely ground 0.3 g samples in concentrated H_2_SO_4_ (Kjeldahl digestion) followed by steam distillation under alkaline conditions and titrimetric determination of the ammonium-N collected in boric acid. Total carbon measurements were performed by dry heating (ashing) at 530°C for 8 h. First, the organic matter content was estimated by measuring the weight loss of the sample following the heating process, whereas total carbon was calculated by multiplying the organic content with a factor of 0.58. Acid detergent fiber (ADF), lignin, and cellulose were determined by the method of van Soest and Wine [35], while hemicellulose was estimated as the difference between ENDF (neutral detergent fiber with enzyme modification) and ADF [36, 37]. The rest of the analyses (unless otherwise specified) were performed as described by Ntougias et al. [38].

For measurements of total phenolics content, cultivation substrates and mushroom samples were freeze-dried and then powders were analyzed by the Folin-Ciocalteu method [39]. Absorbance was measured at 750 nm by using a U-2001 spectrophotometer (Hitachi Instruments Inc., USA). Gallic acid was used as standard for quantification.

 For establishing DPPH scavenging capacity, freeze-dried mushroom powders were mixed with 1 mL methanol, vortexed for 5 min, and then centrifuged for 10 min at 12000 rpm. The antioxidant activity of supernatants was measured by using a 1 mM DPPH (2,2-diphenyl-1-picrylhydrazyl) solution as previously described [40] and by determining absorbance at 515 nm after 30 min incubation.

 The seed germination index (GI) was estimated according to Zucconi et al. [41] protocol by using 25 cress (*Lepidium sativum* L.) seeds per sample; these were placed onto a filter paper moistened either with the sample (1 : 9 TPOMW : water, v/v) or with water (control, GI: 100%), and they were incubated in a Petri dish for 3 d in the dark.

### 2.6. Data Analysis

All experiments were performed in triplicate unless otherwise stated. Results are expressed as mean values ± standard errors. Analysis of variance was conducted by Gabriel's *t*-test at 5% level of probability to compare mean values. Pearson's correlation coefficient was employed to analyze relationships between variables. The SPSS (ver. 18) software was used for all statistical analyses.

## 3. Results

### 3.1. Composting of TPOMW

**Figure 1:**
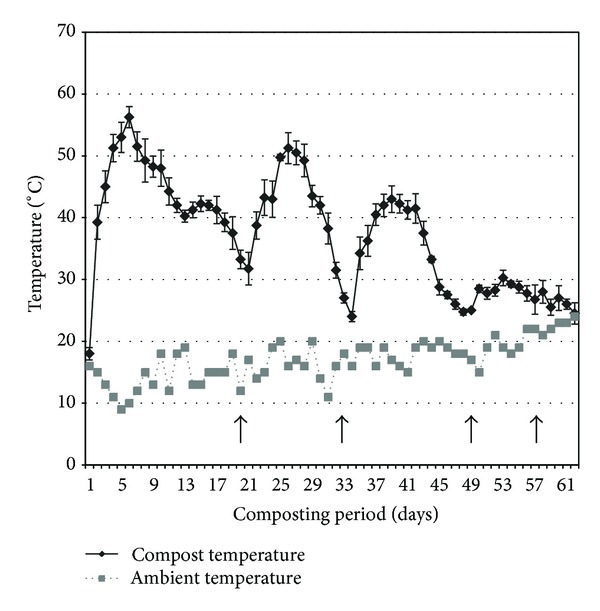
Temperature fluctuations during the TPOMW composting process in the compost pile and in the surrounding environment. *Y*-axis values represent temperatures and *X*-axis values represent measurements taken during the composting process and for a period of 60 days. Vertical arrows indicate the time of pile-turning interventions.

Composting of the TPOMW material exhibited a rather sharp increase in temperature values reaching levels of 57°C within one-two weeks from the start of the process (Figure 1). The first thermophilic phase lasted ca. three weeks, and it was followed by the first turning. Better aeration conditions combined with a higher availability of nutrients led to another rise in temperature at values exceeding 50°C (after one week from turning) and to a second thermophilic phase which lasted for 15 days. The second turning was performed at about the end of the fifth week and was followed by another increase in temperature at ca. 43°C. The last thermophilic phase had a duration of approximately two weeks, and it was followed by the third turning at the end of the seventh week; this intervention resulted in a low temperature raise (30°C), and then a fourth and final turning was performed without any effect in the temperature of the pile indicating thus the end of the process. On the other hand, pH values presented a fast increase from 5.4 to 8.1 within the first four-five weeks of the composting period, and were then stabilized at levels of 8.2–8.3 for the rest of the process (Table 1). Electrical conductivity rose slightly (1.68 mS cm^−1^) as anticipated in the course of composting, whereas organic matter decreased by less than 10% (reaching values of approximately 88%). In addition, C : N ratio decreased considerably (i.e., 22) taking into account that no external nitrogen supplementation was provided during the process. Similarly, lignin, hemicellulose, and cellulose contents of raw TPOMW were reduced by ca. 9%, 40%, and 21%, respectively, in the final composted material. Finally, phytotoxicity decreased substantially from very high levels in both raw and prior to composting substrates (GI%: 20–24) to low values (comparable to the water control) for composted TPOMW (GI%: 88). The results of the analyses of raw and composted TPOMW (before and after composting) are presented in Table 1. For all cultivation substrates prepared, their content in C and N (before and after wheat bran amendment) as well as in lignin, hemicellulose, and cellulose was measured prior to inoculation with the selected fungi (Table 2). Noteworthy were the considerably lower values of C : N ratios (in respect to wheat straw) obtained by high TPOMW supplementation especially in the case of composted materials, for example, 80 versus 29. In parallel, lignin and cellulose contents presented also high differences in TPOMW-rich substrates when compared to the control treatment.

### 3.2. Mycelium Growth Rates and Earliness in “Race” Tubes Experiments

Prior to this study screening experiments with several mushroom species (i.e., *Agrocybe cylindracea*, *Auricularia auricula-judae*, *Lentinula edodes*, *Pleurotus cystidiosus*, *P. eryngii*, *P. ostreatus,* and *P. pulmonarius*) were conducted for qualifying those which could colonize TPOMW-based substrates (data not shown). Subsequent results excluded the use of *A. auricula-judae* and *L. edodes* as well as further experimentation with substrates composed entirely of TPOMW or of 80 : 20 (w/w) mixture with WS since the growth of most strains could not be adequately supported. This prescreening process was adopted because of the significant variability in the efficacy demonstrated by different species/strains in colonizing olive mill wastes [13, 42].

 In the frame of the present work, 15 fungal strains assigned to five mushroom species were evaluated for their ability to colonize TPOMW either raw (in different ratios in mixtures with wheat straw) or pretreated (composted) in “race” tubes experiments (Table 3). In all cases, substrates containing composted TPOMW performed notably better than the respective raw TPOMW treatments. In one particular case (*P. cystidiosus*), the latter substrate did not even permit mycelium growth at the 60 : 40 ratio, whereas the composted material provided growth rates values equivalent to those obtained by raw TPOMW in lower concentrations (e.g., 1.22–1.40 and 1.71–1.91 mm day^−1^ in raw TPOMW 40% and 20%, respectively, versus 1.46–1.56 mm day^−1^ in composted TPOMW 60%). In general, mycelium growth values in composted TPOMW were comparable to those measured for raw TPOMW media with lesser content in the effluent. Noteworthy was also that for all nine *P. eryngii*, *P. ostreatus,* and *P. pulmonarius *strains, the composted TPOMW 20% substrate performed significantly better than the straw (control) treatment, while the same observation could be made for *A. cylindracea* strains as well (albeit differences were not statistically significant in this particular case). Raw TPOMW 60% was the worst performing medium in the course of this evaluation.

 As far as the behavior of individual species is concerned, *P. cystidiosus* strains were the poorest performers by presenting growth rates as low as 1.22 mm day^−1^ or no growth at all at raw TPOMW (40 : 60 and 60 : 40 mixtures, resp.). On the other hand, the fastest colonizers were *P. pulmonarius *and *P. ostreatus*; they performed significantly better than the rest of the species examined in all substrates tested by presenting values as high as 10.02–10.64 and 8.93–10.67 mm day^−1^, respectively, in the composted TPOMW 20 : 80 medium. Furthermore, *P. eryngii *provided relatively high growth rates in all TPOMW containing substrates. Of special interest was the fact that both 20 : 80 and 40 : 60 composted TPOMW ratios provided significantly better growth rates than the straw substrate (7.28–8.72 versus 5.58–6.42 mm day^−1^, resp.). Lastly,  *A. cylindracea* mycelia supported well increased concentrations of TPOMW and even when grown at the 60 : 40 composted TPOWM medium, the values obtained were slightly lower than those from the control treatment (3.81–4.62 versus 4.35–5.38 mm day^−1^, resp.).

**Figure 2:**
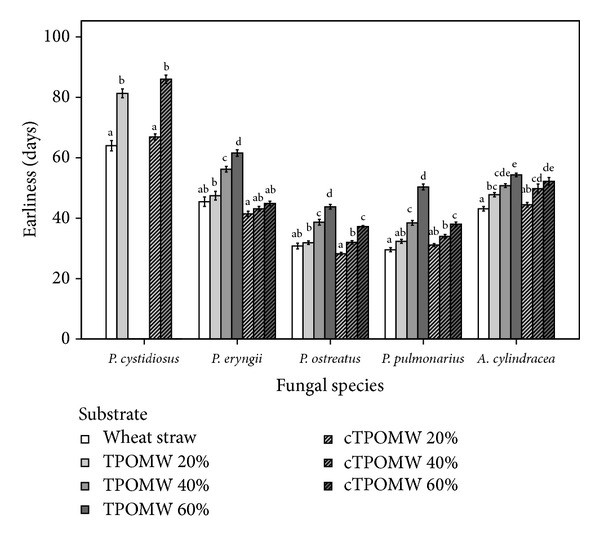
Earliness (time period elapsed from substrate inoculation until the appearance of basidiomata primordia) for five mushroom species colonizing seven substrates in “race-tube” experiments. Values (days) from three strains per species were pooled and results are expressed as means ± standard errors of means, *n* = 9. Lack of letters in common indicates statistically significant differences (Gabriel's *t*-test, *P* < 0.05) among substrates for each species examined.

 Within the frame of the same experiment, the time period needed from substrate inoculation until mushroom initial formation was noted (Figure 2). *P. ostreatus* and *P. pulmonarius* strains were the first to form primordia in all substrates within a period of 25 to 40 days (with the exception of raw TPOMW 60% where slightly longer periods were required). *P. eryngii* and *A. cylindracea *needed 40 to 60 days for primordia formation, whereas *P. cystidiosus* was significantly slower (in line with the low growth rates it produced) since it needed 60 to 90 days to produce mushroom initials. Statistical comparisons among different substrates for the same species demonstrated that wheat straw (control) and composted TPOWM 20%, were the most suitable media for fast primordia formation in *P. cystidiosus* and *A. cylindracea*, while wheat straw (control), raw and composted TPOWM 20%, and composted TPOMW 40% provided the best results for *P. eryngii*, *P. ostreatus, *and *P. pulmonarius*.

### 3.3. TPOMW-Based Substrates Evaluated for Mushroom Cultivation

The results of the “race-tubes” experiment permitted the qualification of four (the best performing) strains from four out of the five species initially examined. Hence, with the exception of all *P. cystidiosus* strains which were considerably slower and failed to yield consistently mushroom primordia, the following strains were further evaluated in mushroom cultivation trials: *P. eryngii* LGAM101, *P. ostreatus* LGAM60, *P. pulmonarius* LGAM10, and *A. cylindracea* LGAM281. In addition, all six TPOMW containing substrates were further examined as regards their suitability to support mushroom production in comparison to the wheat-straw (control) substrate.

 Strains of *P. ostreatus* and *P. pulmonarius* were those that formed mushroom primordia within the shortest time period after substrate inoculation (Table 4). Hence, their respective earliness values were 4 to 5 weeks, such short periods being observed when fungi colonized either the wheat straw or composted TPOMW 20% substrates (26–30 days). However, these values for both *P. ostreatus* and *P. pulmonarius* were not significantly different from those noted in the other substrates examined (with the exception of raw TPOMW 60%). In contrast, earliness for the other two species *P. eryngii* and *A. cylindracea* was significantly higher ranging from 37–47 days for the former to 38–44 days for the latter species. Again, wheat straw and composted TPOMW 20% and 40% substrates provided lower values in comparisons among different substrates for the same fungus albeit differences were not statistically significant.

**Figure 3:**
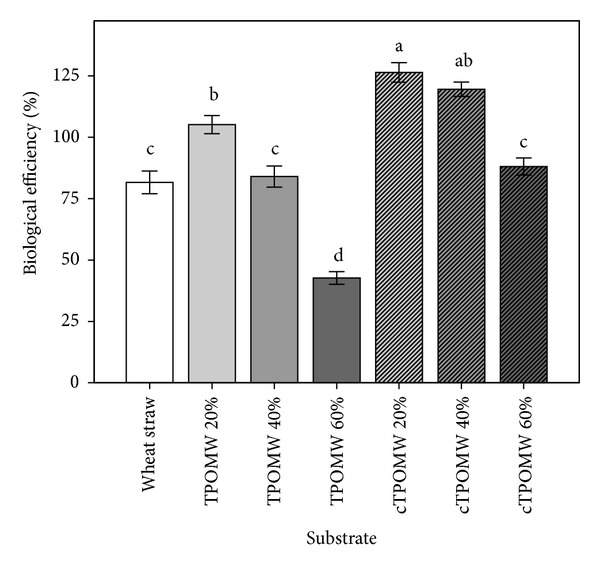
Biological efficiency (percentage ratio of fresh mushrooms weight over the dry weight of the respective substrate) for four mushroom species growing on seven cultivation substrates (TPOMW: raw two-phase olive mill waste; cTPOMW: composted two-phase olive mill waste). Values (%) from four species were pooled and results are expressed as means ± standard errors of means, *n* = 12. Lack of letters in common indicates statistically significant differences (Gabriel's *t*-test, *P* < 0.05) among the substrates examined.

 The cropping period lasted 23–46 days for *P. eryngii* and *A. cylindracea* (two production flushes were obtained) and 30–52 days for *P. ostreatus* and *P. pulmonarius* (three production flushes). For *P. eryngii*, total yields ranged from 93 to 364 g with the respective BEs ranging from 31 to 120 (Table 4; Figure 3). Despite the fact that most comparisons among different substrates did not result in statistical significant differences due to the relatively high variation of means, the composted TPOMW 20% and 40% provided very high values in respect to most other treatments including the control (BEs: 120 versus 88). In contrast, raw TPOMW 60% exhibited the lowest values within the lengthier cropping period (BE: 33 in 46 days as opposed to cropping periods of 23 days for composted TPOMW 20%). On the other hand, *P. ostreatus* demonstrated yields of up to 410 g (BE: 135) in the composted TPOMW 20% treatment which almost doubled the respective value provided by the control treatment (i.e., 213 g, BE: 70). This particular substrate as well as composted TPOMW 40% performed significantly better than the rest examined for *P. ostreatus*; it is noteworthy that the wheat straw supported the lowest yield values among all substrates examined (except of the raw TPOMW 60%) for this particular species albeit within a relatively shorter period. In addition, *P. pulmonarius* provided also its highest yields in the composted TPOMW 20% (381 g and BE: 126); however, composted TPOMW 40% and raw TPOMW 20% presented also high and comparable BE values (116-117), although not significantly different from the control treatment (100). Only the raw TPOMW 60% substrate performed significantly worse than wheat straw. Lastly,  *A. cylindracea* demonstrated an exceptionally good adaptation to olive mill waste containing substrates since all TPOMW media (except of raw TPOMW 60%) provided significantly higher yields and BE values in comparison to the control, that is, 291–378 g and BEs: 96–125 versus 210 g and BE: 69.

**Figure 4:**
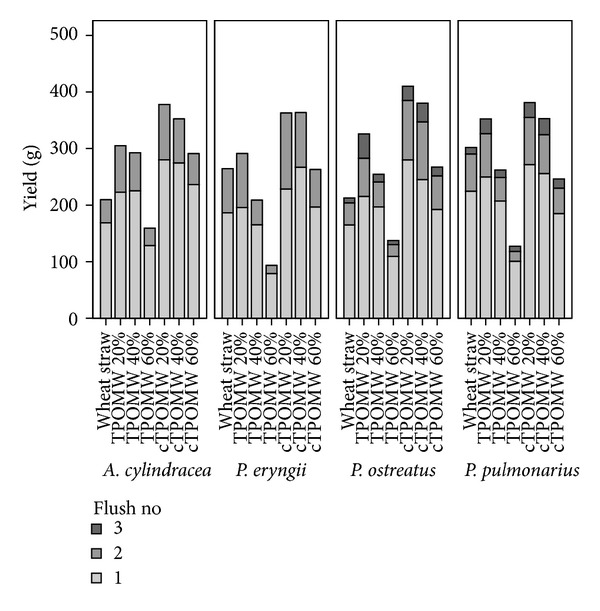
Distribution of mushroom yield per production flush for four mushroom species growing on seven cultivation substrates (TPOMW: raw two-phase olive mill waste; cTPOMW: composted two-phase olive mill waste). Values (g) are expressed as means, *n* = 3.

 As regards the distribution of yields per flush (Figure 4), *P. eryngii* and *A. cylindracea* exhibited a tendency to produce a larger part of their production in the first flush as the relative content of TPOMW increased in the substrate's composition; for example, for *A. cylindracea* 81% of the total production was harvested from the first flush in the composted TPOMW 60% as opposed to 74% in the composted TPOMW 20% substrate (77% and 73% for the raw TPOMW 40% and 20% substrates, resp.). In the three-flush harvested species (*P. ostreatus* and *P. pulmonarius*), approximately 10% (or less) of the total yield was obtained from the third flush, whereas the first flush generally contributed 65–75% of the total yield. Again, although not as evident as before the two of the richest in TPOMW substrates (raw 40% and composted 60%) produced a first flush production which accounted for 72–79% of the total in both species.

 Substrates and fungal strains were also evaluated with respect to the weight of mushrooms obtained. *P. eryngii* produced the heaviest mushrooms among all species tested and this is in accordance to the habit of this particular fungus to form single basidiomata whereas the other three species form clusters composed of many basidiomata, each. Weight of individual *P. eryngii* mushrooms were significantly higher in the control treatment (27 g) followed by all other treatments which showed similar results (i.e., from 16 to 18 g) (Table 4). In the cases of *P. ostreatus* and *P. pulmonarius* the size of mushrooms was not significantly different in comparisons between all treatments for the same species, and hence the nature of the substrate did not seem to exert any particular influence. *A. cylindracea* basidiomata were similar in size when different TPOMW-based media were compared but again the control treatment supported the growth of larger mushrooms. Moreover, in comparisons performed among different species, *P. ostreatus* mushrooms produced from substrates composed of TPOMW (raw and composted) were heavier than those of *P. pulmonarius* and *A. cylindracea*.

**Figure 5:**
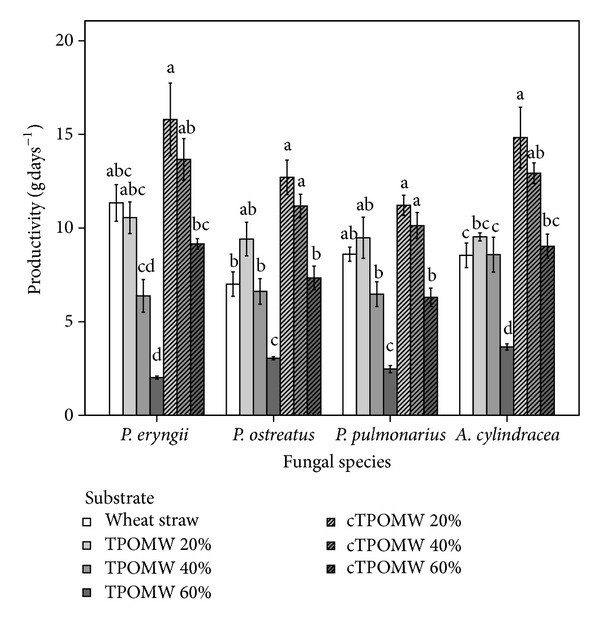
Productivity (ratio of total yield over the duration of the cropping period) for four mushroom species growing on seven cultivation substrates (TPOMW: raw two-phase olive mill waste; cTPOMW: composted two-phase olive mill waste). Values (g days^−1^) are expressed as means ± standard errors of means, *n* = 3. Lack of letters in common indicates statistically significant differences (Gabriel's *t*-test, *P* < 0.05) among substrates for each species examined.

 Another cultivation parameter evaluated was productivity, which was defined as total mushroom yield produced within the particular cropping period for each substrate and species examined (Figure 5). The highest productivity was exhibited by the composted TPOMW 20% and 40% media, albeit not statistically significant in most cases. These two particular substrates supported high yields within relatively short time frames. Of importance was that the control medium (wheat straw) was notably inferior in productivity than composted TPOMW 20% by ca. three times in the case of *A. cylindracea* (statistically significant difference) and by almost two times in the case of *P. ostreatus*. As regards *A. cylindracea* especially, all TPOWM amended substrates (with the exception of raw TPOMW 60%) performed significantly better than the control. As regards *Pleurotus* spp., raw TPOMW 40% and composted TPOMW 60% presented relatively inferior productivity values than the rest of the treatments, whereas raw TPOMW 20% performed equally well or even slightly better (*P. ostreatus*) than wheat straw. Lastly, raw TPOMW 60% performed poorly.

 The total phenolic content and antioxidant activity of the four strains examined were not significantly different among the various TPOMW substrates examined ranging from 0.10 to 0.17 mg mL^−1^ gallic acid equivalents (raw TPOMW 40% and composted TPOMW 60% presenting the highest contents). Similarly, the DPPH scavenging capacity did not differ significantly among cultivation substrates; however, the *P. eryngii *strain demonstrated higher antioxidant values reaching up to 84% (compared to *A. cylindracea*'s 59%, *P. ostreatus*'s 68%, and *P. pulmonarius*'s 72%). 

## 4. Discussion

For all strains tested, mycelium grew equally well or (in most cases) significantly better in composted TPOMW than in noncomposted substrates (in comparisons made between equal rates of supplementation in respect to wheat-straw). Similarly, and most importantly still, addition of composted TPOMW 20% and 40% into the growth substrate resulted in high mycelium growth rates accompanied by notably better earliness values in respect to the control (wheat straw). It is evident that the TPOMW pretreatment through the composting process alleviated (at least in part) the toxicity of the effluent while at the same time provided readily available nutrients through the action of the thermophilic microbiota as it was also demonstrated by previous studies on TPOMW composting [3, 5, 8, 43]. Indeed, phytotoxicity tests performed with both raw and composted TPOMW demonstrated that toxicity of the latter was significantly reduced. Moreover, composting might have also ensured the presence of inducer compounds, which especially in the case of the white-rot fungi enhance the activity of their lignin-degrading system [44, 45]. In previous pertinent studies, the use of OMWW either as ingredient or just as wetting agent of *Pleurotus* cultivation media [16–18] contributed at maintaining pH values at levels close to those commonly used for its commercial production (i.e., 6.0–7.0). This work revealed that, although composted TPOMW substrates possessed a higher pH (i.e., 7.5–8.0), satisfactory growth of mycelia and high mushroom yields were achieved vis-à-vis raw TPOMW and wheat-straw media with lower pH values. Similar observations on the successful use of other mushroom substrates with pH values of up to 7.5 were also made by Yildiz et al. [46].

 The “race” tubes evaluation excluded the subsequent use of one species (*P. cystidiosus*) due to the significantly slower colonization rates it demonstrated. In addition, eight out of the twelve strains initially screened for the rest of the four species examined were also abandoned. Hence, cultivation trials were further performed with the four most efficient (faster growing and earlier in producing mushroom primordia) strains of *A. cylindracea*, *P. eryngii*, *P. ostreatus,* and *P. pulmonarius* on seven cultivation substrates. In general, higher TPOMW supplementation resulted in longer colonization periods despite the fact that differences noted for earliness were not statistically significant. However, similar effects were also reported in the past when various types of olive mill wastes were added to *Pleurotus* cultivation substrates resulting in delayed formation of primordia [17, 18, 20]. In all these cases, increase in olive waste amendment has a distinct adverse effect on earliness; interestingly enough, this seems to be (at least partly) mitigated when composted TPOMW was used instead. Furthermore, earliness values observed in this work were much lower than those reported when *P. eryngii* and *P. pulmonarius* were examined on extracted olive press cake supplemented with OMWW [18] or when *A. cylindracea* was examined on cotton waste, wheat straw, and peanut shells [25]. On the other hand, they are similar to those reported on raw TPOMW for *P. ostreatus* and *P. pulmonarius* [20] and on cotton waste, wheat straw and peanut shells for *P. ostreatus*, *P. pulmonarius,* and *P. eryngii* [25].

 TPOMW-based substrates supported high yields for all species examined. The highest BE values were obtained from the composted TPOMW 20% medium, which performed distinctly better for all species examined in comparison with the control treatment (albeit differences were not always statistically significant due to high errors of the means). Almost equally satisfactory was the performance of raw TPOMW 20% and composted TPOMW 40% substrates, whereas even in higher TPOMW amended treatments (i.e., raw TPOMW 40% and composted TPOMW 60%) yields were comparable to those of the control, and only the raw TPOMW 60% underperformed. The values obtained are markedly higher than those reported in the literature. In the only previous study on the use of raw TPOMW as oyster mushroom cultivation substrate [20], BE values of as high as 85% were observed in substrates containing 50% effluent, whereas at higher concentrations they decreased rather sharply (64% at TPOMW 60%). Values reported in the present work are considerably higher as regards the performance at lower raw TPOMW concentrations (i.e., 20% and 40%) but especially when composted media were used. However, results of both studies seem to coincide as concerns the decrease in mushroom yields that becomes significant at levels of TPOMW supplementation exceeding 50%.

 Moreover, BEs noted in the present study are higher than most of those obtained through the use of other substrates. In the past, *P. eryngii* production on either supplemented [25, 27, 47] or amended with a casing layer [48, 49] substrates presented BEs of 7–88% and 47–115%, respectively. The outcome of the present work revealed that addition of composted TPOMW enhanced considerably the performance of wheat straw based substrates by reaching BE values of 120%. More importantly, this result was obtained without addition of rich in nitrogen (e.g., soybean flour) and/or delayed-release nutrients and without the use of any casing material which when applied is reported to increase significantly yields in *P. eryngii* cultivation [49, 50].

 Similarly, *P. ostreatus* and *P. pulmonarius* yields in TPOMW amended substrates were very high and in the case of the former species they almost doubled those measured in wheat straw. Both strains presented BEs exceeding 100% in three different TPOMW media (raw 20%, and composted 20% and 40%), that is, 108–135%. BEs reported in previous studies demonstrated a high variability depending on the nature of substrate, type of supplementation, and the strain(s) used; still, the respective values reported are in most cases lower than those achieved through the use of TPOMW containing media. Hence, for *P. ostreatus*, values ranged from 0% to 96% in wheat straw amended with solid waste from anaerobic digestion of litter from broiler chickens, 14% in peanut shells, 4–61% in different types of sawdust, 8–46% in wheat straw wetted by olive mill wastewater, 37–79% in vineyard pruning and grape pomace, 50–94% in wheat straw, 72–87% in coffee pulp, to 71–117% in cotton wastes [16, 24, 25, 51–53]. It is of interest that even when a substrate containing sunflower seed hulls received supplementation with optimized levels of Mn(II) and/or NH_4_, BE values for *P. ostreatus* did not exceed 112% [54]. As regards *P. pulmonarius*, the BEs ranged from 19% in peanut shells, 38–63% in vineyard pruning and grape pomace, 50–81% in coffee pulp, 93–97% in cotton waste, to 66–123% in wheat straw [25, 51, 52].

 Most of the cultivation studies conducted so far on *A. cylindracea* focused on the use of wheat straw usually supplemented with various nitrogen-rich materials, while alternative substrates were only occasionally evaluated, for example, willow sawdust, poultry litter, cotton waste, and peanut shells [25, 29, 55]. In general, amended wheat straw based media supported high yields either when soybean flour was added (BE: 138–179% within 53–64 days of cropping period corresponding to three production flushes) [29] or when solid waste from anaerobic digestion of poultry litter was used (BE: 106% over a 115-day fruiting cycle corresponding to five flushes) [55]. On the other hand, this work evaluated for the first time the potential use of olive mill wastes as substrate for *A. cylindracea* cultivation; both raw and composted TPOMW (20% and 40%) based media exhibited significantly higher BE than the wheat straw control ranging from 97% to 125% within a cropping period of 26 to 34 days including two production flushes only. 

 Substrate supplementation represents a key issue in developing successful solid-state fermentation of various lignocellulosic materials for mushroom cultivation. In the past, the amendment of straw-based *Pleurotus* spp. and *A. cylindracea* substrates with different nitrogen-rich compounds received much attention [27, 29, 54, 56]. Despite the fact that addition of such materials clearly results in higher yields, their use is also linked with increased possibility of compromising mushroom production due to the fast rise in substrate temperature because of enhanced fungal metabolic activities triggered by the presence of extra nitrogen [56, 57] and/or to the significantly higher contamination risks by competitor microorganisms [46, 58].

 If TPOMW is considered as a supplement to conventional wheat straw substrates, the evaluation of its effects on mushroom production demonstrates that its presence at rather low concentrations (i.e., 20% w/w) significantly enhanced BE, while it had no effect on earliness, size of basidiomata, and quality traits (phenolic and antioxidants content, shape of mushrooms). Furthermore, it seems that the presence of composted TPOMW promotes high mushroom yields through the enrichment of the cultivation media with several macro- and micronutrients which are otherwise not provided (in the conventional wheat straw substrates). More interestingly still this type of supplementation is considered advantageous as opposed to that provided by other nitrogen rich compounds, which are ordinarily used as amendments in mushroom cultivation (e.g., soybean flour). Although this latter type of material provides readily assimilated forms of nutrients whose impacts are easily identifiable on the performance of the subsequent mushroom crop, at the same time its use presents significant drawbacks as previously explained. In contrast, a TPOMW-based cultivation medium maintains most of the inherent advantageous properties of a typical plain lignocellulosic substrate for the selective growth of mushroom species [59]. Since it possesses a rather high content in complex organic compounds, it provides a very important competitive advantage for the cultivated mushroom, especially under commercial cultivation conditions. Along the same line of arguments, Altieri et al. [19] used a substrate mainly composed of destined olive husk taken from two-phase olive mills for commercial-scale cultivation of *Agaricus bisporus* and demonstrated that this material presented improved selectivity and hence better protection against competitors. At the same time it possessed higher content in organic nitrogen and enhanced organic matter degradation in comparison to the conventional wheat straw plus chicken manure compost.

 Correlation of substrates contents versus cultivation parameters examined in the frame of the present study revealed that substrates hemicellulose content was negatively correlated with mycelium growth rates and BEs (*r* ^2^: 0.75–0.91 and 0.59–0.88, resp.) and positively with earliness (*r* ^2^: 0.81–0.95). Furthermore, lignin content presented a negative correlation with earliness (*r* ^2^: 0.80–0.94) in contrast to what was observed for cellulose : lignin ratio (*r* ^2^: 0.60–0.83). Lastly, C : N and cellulose : lignin ratios revealed a positive correlation with mushroom weight for *P. eryngii* and *A. cylindracea* strains (*r* ^2^: 0.83–0.85 and 0.84–0.85, resp.). As regards *A. cylindracea* especially, cellulose : lignin ratio was positively correlated with mycelium growth rate which is in accordance with earlier observations on other cultivation substrates indicating that this species preferentially consumes cellulose [25, 55]. It is worth mentioning that C : N ratio was not correlated with mycelium growth rates and BEs as it was reported in a previous study [25], and this could be attributed to the fact that although increased TPOMW supplementation results in higher N (and other nutrients) content, at the same time the higher toxicity of this material has adverse effects which progressively eliminate its positive influence. This was evidenced when the raw TPOMW 60% treatment was excluded from the correlation analysis, which resulted at obtaining relatively high correlation values for C : N versus growth rates and BEs (e.g., 0.63–0.68 and 0.77–0.92, resp.).

 The issue of the final product quality, especially when mushrooms are cultivated on such a type of substrates is also of importance. In the frame of this study, harvested mushrooms were examined for any deformations or abnormalities in shape or colour due to the TPOMW amendments. Moreover, their content in phenolic compounds was assessed with respect to that the level of TPOMW supplementation of their growing substrate received. As regards their appearance, no deformations were observed apart from the pilei in *Pleurotus* strains, which presented slightly lighter colours in increased TPOMW concentrations (40% and 60% rates). This observation is in accordance with the findings of Ruiz-Rodriguez et al. [20] and might be attributed to hindered production of melanin-related pigments. However, such colour variations in *Pleurotus* pilei are common even among strains of the same species, while they also appear under the influence of different environmental parameters [22]. Hence, they do not constitute an adverse quality trait for the product itself or for its acceptance by the market. Most importantly, the total phenolic content and antioxidant activity values were not statistically different in comparisons among straw and TPOMW based substrates indicating thus that no inhibition of oxidative enzymes or absorption of phenol-related compounds occurred. These results confirm and expand the conclusions drawn by a previous pertinent study employing *P. ostreatus* and *P. pulmonarius* strains [20]. 

 Moreover, the mushroom cultivation process developed on TPOMW-based media, apart from providing an edible product of high added value, can also lead to the exploitation of the spent substrate. Treatment of TPOMW with mushroom fungi could constitute an alternative economical method in order to convert olive wastes into valuable amendments for agricultural soils or constituents of animal feed. The final by-product has a low content in phenolic compounds through the action of fungal ligninolytic enzymes leading to a significant decrease of biotoxicity [13, 38, 60], while it presents lower values in total organic carbon, humic acids and lignocellulosic compounds, lower C : N ratio, and increased content in nutrients including proteins [19, 61, 62].

 In conclusion, productivity of all four mushroom species examined was found to be the highest when wheat straw (the basic component for most mushroom production substrates) was supplemented with 20–40% composted TPOMW (or 20% raw TPOMW). Only when supplementation exceeded 60% for raw TPOMW, a negative impact was noted on mushroom yields, which could be attributed to the biotoxic effect of the effluent. In fact, the difference observed in comparisons between raw and composted TPOMW is directly related with the composting process itself since material subjected to the latter type of treatment was significantly detoxified as the phytotoxicity tests revealed. Hence, TPOMW-based media are promising alternative substrates for the cultivation of *Pleurotus *spp. and *A. cylindracea*. The production process is in need of relatively minor modifications aiming mainly at reducing the incubation period and the rather high variability observed in mushroom productivity. In addition, of importance is the exploitation of a waste material which until now is associated with significant problems regarding its safe environmental disposal. Moreover, the valorization of new substrates for the cultivation of choice mushroom species (some of which are still not widely cultivated in Europe and hence their commercial production could lead to a much-sought after market diversification) is of further interest.

## Figures and Tables

**Table 1:** Properties of raw two-phase olive mill waste (TPOMW), of TPOMW (plus olives leaves) prior to composting, and of TPOMW after composting.

	Raw TPOMW	TPOMW prior to composting	TPOMW after composting
pH	5.1	5.92	8.15
EC^1^ (mS cm^−1^)	1.08	1.15	1.68
Total C (%)	53.64	51.07	42.47
Total N (%)	1.31	1.45	1.96
C : N	40.95	35.22	21.67
TOM^2^ (%)	96.21	94.31	87.88
Ash (%)	3.47	4.12	4.76
P (g kg^−1^)	1.01	1.08	1.23
K (g kg^−1^)	15.4	16.3	21.34
Lignin (g kg^−1^)	351	343	318
Hemicellulose (g kg^−1^)	380	341	226
Cellulose (g kg^−1^)	211	208	167
GI^3^ (%)	20	24	88

^1^EC: electrical conductivity.

^2^TOM: total organic matter.

^3^GI: germination index.

**Table 2:** Total carbon (C), nitrogen (N), lignin, hemicellulose, and cellulose contents (values expressed on a dry matter basis) of the substrates used for mushroom cultivation experiments.

Substrates^1^	C^2^ (% d.w.)	N^2^ (% d.w.)	C : N^2^	C : N^3^	Lignin^2^ (g kg^−1^)	Hemicellulose^2^ (g kg^−1^)	Cellulose^2^ (g kg^−1^)
WS	44.15	0.43	102.67	79.78	191	218	370
TPOMW 20%	49.98	0.72	69.42	60.44	224	242	334
TPOMW 40%	47.79	0.89	53.70	46.12	261	288	305
TPOMW 60%	44.12	0.99	44.57	37.57	296	317	271
cTPOMW 20%	42.02	0.93	45.18	40.55	221	220	321
cTPOMW 40%	40.34	1.05	38.42	35.46	250	222	287
cTPOMW 60%	37.76	1.24	30.45	28.74	273	223	231

^1^WS: wheat straw; TPOMW: raw two-phase olive mill waste; cTPOMW: composted two-phase olive mill waste. Percentage values following TPOMW refer to its respective content (w/w) in the cultivation substrate.

^2^Values for substrates prior to any wheat bran amendment.

^3^Values after amendment of wheat bran.

**Table 3:** Mycelium growth rates of 15 strains belonging to five mushroom species (P.cs: *Pleurotus cystidiosus*; P.er.: *P. eryngii*; P.os: *P. ostreatus*; P.pl: *P. pulmonarius*; A.cl: *Agrocybe cylindracea*) colonizing seven substrates (WS: wheat straw; TPOMW: raw two-phase olive mill waste; cTPOMW: composted two-phase olive mill waste) in “race-tube” experiments. Values (mm/day) are expressed as means ± standard errors of means, *n* = 3. Lack of letters in common indicates statistically significant differences (Gabriel's *t*-test, *P* < 0.05) for comparisons of treatment means between different strains (capital letters) and different substrates (lowercase letters).

Species	Strain	WS	TPOMW 20%	TPOMW 40%	TPOMW 60%	cTPOMW 20%	cTPOMW 40%	cTPOMW 60%
P.cs.	LGAM50	2.28 ± 0.21^a.F^	1.74 ± 0.13^bcd.F^	1.22 ± 0.07^d.G^	nd	2.20 ± 0.07^ab.G^	1.94 ± 0.05^abc.K^	1.46 ± 0.05^cd.G^
	LGAM100	1.99 ± 0.12^ab.F^	1.71 ± 0.07^abc.F^	1.40 ± 0.03^c.G^	nd	2.14 ± 0.09^a.G^	2.05 ± 0.11^a.K^	1.56 ± 0.12^bc.G^
	D415	2.55 ± 0.10^a.F^	1.91 ± 0.07^bc.F^	1.31 ± 0.06^e.G^	nd	2.23 ± 0.11 ^ab.G^	1.79 ± 0.08^cd.K^	1.47 ± 0.06^de.G^

P.er.	LGAM101	6.42 ± 0.36^cd.CD^	7.49 ± 0.32^bc.CD^	6.46 ± 0.21^cd.D^	5.38 ± 0.21^d.D^	8.72 ± 0.12^a.D^	8.39 ± 0.08^ab.EF^	7.45 ± 0.16^bc.C^
	LGAM63	6.26 ± 0.24^d.CD^	7.26 ± 0.14^bc.D^	6.42 ± 0.20^d.D^	5.05 ± 0.17^e.D^	8.51 ± 0.08^a.D^	7.93 ± 0.08^ab.F^	7.05 ± 0.12^cd.CD^
	UPA10	5.58 ± 0.30^c.CD^	6.73 ± 0.16^b.D^	5.37 ± 0.13^c.E^	5.37 ± 0.28^c.D^	7.73 ± 0.16^a.E^	7.28 ± 0.14^ab.G^	6.61 ± 0.10^b.D^

P.os.	LGAM60	8.96 ± 0.28^d.A^	9.94 ± 0.27^abc.A^	9.06 ± 0.10^cd.A^	7.68 ± 0.17^e.AB^	10.67 ± 0.11^a.A^	10.15 ± 0.15^ab.A^	9.29 ± 0.08^bcd.A^
	LGM850402	7.51 ± 0.31^c.BC^	7.66 ± 0.17^bc.CD^	7.31 ± 0.21^cd.C^	6.38 ± 0.15^d.C^	8.93 ± 0.13^a.D^	8.82 ± 0.14^a.DE^	8.55 ± 0.07^ab.B^
	LGAM106	8.13 ± 0.36^bcd.AB^	8.61 ± 0.37^abc.BC^	7.87 ± 0.10^cd.BC^	7.07 ± 0.19^d.BC^	9.70 ± 0.09^a.C^	9.03 ± 0.05^ab.CD^	8.49 ± 0.09^bc.B^

P.pl.	LGAM26	8.90 ± 0.15^b.A^	9.36 ± 0.25^ab.AB^	8.65 ± 0.26^b.AB^	7.61 ± 0.15^c.AB^	10.04 ± 0.10^a.BC^	9.42 ± 0.11^ab.BC^	8.92 ± 0.11^b.AB^
	LGAM10	9.28 ± 0.29^bc.A^	10.05 ± 0.15^ab.A^	9.01 ± 0.14^c.A^	8.07 ± 0.15^d.A^	10.64 ± 0.16^a.AB^	9.99 ± 0.11^ab.AB^	9.38 ± 0.10^bc.A^
	LGAM580403	9.08 ± 0.13^bc.A^	9.13 ± 0.22^bc.AB^	8.82 ± 0.08^c.A^	7.23 ± 0.17^d.AB^	10.02 ± 0.11^a.BC^	9.68 ± 0.08^ab.AB^	8.89 ± 0.10^c.AB^

A.cl.	IK10	4.35 ± 0.20^bc.E^	4.27 ± 0.20^c.E^	3.36 ± 0.10^d.F^	2.69 ± 0.13^d.E^	5.65 ± 0.15^a.F^	5.11 ± 0.12^ab.HI^	4.32 ± 0.13^c.EF^
	LGAM281	5.38 ± 0.13^ab.DE^	5.04 ± 0.12^bc.E^	3.92 ± 0.11^d.F^	3.18 ± 0.14^e.E^	5.87 ± 0.10^a.F^	5.53 ± 0.10^ab.H^	4.62 ± 0.13^c.E^
	SIEF0834	5.15 ± 0.15^a.DE^	4.80 ± 0.18^a.E^	3.48 ± 0.12^b.F^	2.51 ± 0.10^c.E^	5.36 ± 0.12^a.F^	4.89 ± 0.10^a.I^	3.81 ± 0.14^b.F^

**Table 4:** Data from mushrooms production experiment evaluating cultivation parameters for four selected strains (one per species: P.cs: *Pleurotus cystidiosus*; P.er: *P. eryngii*; P.os: *P. ostreatus*; P.pl: *P. pulmonarius*; A.cl: *Agrocybe cylindracea*) and seven cultivation substrates (WS: wheat straw; TPOMW: raw two-phase olive mill waste; cTPOMW: composted two-phase olive mill waste). Values are expressed as means ± standard errors of means, *n* = 3. Lack of letters in common indicates statistically significant differences (Gabriel's *t*-test, *P* < 0.05) for comparisons of treatment means between different species (capital letters) and for the same species (lowercase letters).

Substrates	Species	Earliness (days)	Cropping period (days)	Yield 1st flush (g)	Yield 2nd flush (g)	Yield 3rd flush (g)	Total yield (g)	BE %	Mushroom weight (g)
WS	P.er.	38.33 ± 1.45^ab.B^	23.33 ± 0.88^a.B^	186.42 ± 13.34^b.AB^	78.04 ± 12.04^bc.A^	nd	264.47 ± 23.89^ab.A^	87.52 ± 7.65^ab.AB^	26.65 ± 1.38^a.A^
	P.os.	26.00 ± 1.16^a.A^	30.67 ± 1.76^a.BC^	164.60 ± 6.28^cd.B^	39.18 ± 2.45^bc.A^	8.87 ± 1.30^cd.A^	212.65 ± 9.93^cd.A^	70.18 ± 3.28^cd.B^	6.01 ± 0.31^a.B^
	P.pl.	29.33 ± 1.20^a.A^	35.00 ± 1.53^a.C^	224.33 ± 13.44^ab.A^	65.97 ± 9.25^ab.A^	11.68 ± 1.90^c.A^	301.97 ± 24.52^abc.A^	99.66 ± 8.09^abc.A^	4.20 ± 0.32^a.B^
	A.cl.	37.67 ± 1.45^a.AB^	24.67 ± 0.88^a.AB^	168.75 ± 5.25^bc.B^	40.86 ± 8.21^cd.A^	nd	209.61 ± 8.34^bc.A^	69.17 ± 2.75^bc.B^	3.36 ± 0.20^a.B^

TPOMW 20%	P.er.	43.00 ± 1.00^bc.B^	27.67 ± 1.67^ab.A^	195.72 ± 13.55^ab.A^	95.54 ± 11.07^b.A^	nd	291.25 ± 24.17^ab.A^	96.12 ± 7.98^ab.A^	18.32 ± 1.17^b.A^
	P.os.	31.00 ± 1.16^ab.A^	35.00 ± 2.08^ab.AB^	215.31 ± 13.05^abc.A^	67.38 ± 6.61^b.A^	43.21 ± 6.46^a.A^	325.90 ± 15.87^ab.A^	107.56 ± 5.24^ab.A^	5.71 ± 0.17^a.B^
	P.pl.	34.33 ± 1.20^ab.A^	37.67 ± 2.19^a.B^	249.61 ± 15.66^ab.A^	76.55 ± 6.48^a.A^	26.36 ± 1.53^ab.B^	352.52 ± 23.33^ab.A^	116.34 ± 7.70^ab.A^	3.41 ± 0.21^a.BC^
	A.cl.	43.67 ± 0.88^b.B^	32.00 ± 1.53^bc.AB^	222.71 ± 22.46^ab.A^	82.32 ± 7.60^ab.A^	nd	305.03 ± 16.97^a.A^	100.67 ± 5.60^a.A^	2.48 ± 0.14^b.C^

TPOMW 40%	P.er.	47.33 ± 1.20^c.B^	33.00 ± 1.00^b.A^	164.99 ± 18.85^b.A^	43.81 ± 3.67^cd.A^	nd	208.80 ± 22.31^b.A^	68.91 ± 7.36^b.A^	15.96 ± 0.42^bc.A^
	P.os.	34.33 ± 0.88^b.A^	38.67 ± 0.88^bc.BC^	196.73 ± 12.79^bc.A^	44.11 ± 6.75^bc.A^	13.84 ± 1.86^cd.A^	254.68 ± 20.26^bc.A^	84.05 ± 6.69^bc.A^	5.92 ± 0.18^a.B^
	P.pl.	34.33 ± 1.20^ab.A^	40.67 ± 1.20^a.C^	207.24 ± 19.69^ab.A^	41.67 ± 3.14^bc.A^	13.00 ± 1.98^c.A^	261.92 ± 21.95^bc.A^	86.44 ± 7.24^bc.A^	3.95 ± 0.10^a.C^
	A.cl.	42.33 ± 0.88^b.B^	34.33 ± 1.20^c.AB^	225.09 ± 16.26^ab.A^	67.57 ± 8.73^abc.A^	nd	292.65 ± 23.56^ab.A^	96.58 ± 7.77^ab.A^	2.35 ± 0.06^b.D^

TPOMW 60%	P.er.	55.67 ± 0.88^d.B^	46.33 ± 0.88^c.A^	78.73 ± 4.51^c.B^	14.76 ± 1.05^d.B^	nd	93.49 ± 5.56^c.B^	30.85 ± 1.84^c.B^	13.50 ± 0.17^c.A^
	P.os.	40.33 ± 0.88^c.A^	45.00 ± 1.16^c.A^	109.44 ± 7.26^d.A^	20.63 ± 1.11^c.B^	7.38 ± 1.26^d.A^	137.46 ± 7.18^d.A^	45.37 ± 2.37^d.A^	5.20 ± 1.16^a.B^
	P.pl.	43.67 ± 1.20^c.A^	51.67 ± 1.20^b.B^	100.55 ± 7.22^c.AB^	17.40 ± 2.04^c.B^	9.41 ± 0.91^c.A^	127.35 ± 7.77^d.AB^	42.03 ± 2.56^d.AB^	3.39 ± 0.08^a.C^
	A.cl.	52.67 ± 0.88^c.B^	43.67 ± 0.88^d.A^	128.41 ± 6.03^c.A^	31.07 ± 1.93^d.A^	nd	159.48 ± 7.96^c.A^	52.63 ± 2.63^c.A^	1.99 ± 0.07^b.D^

cTPOMW 20%	P.er.	36.67 ± 0.33^a.B^	23.33 ± 1.45^a.A^	228.10 ± 16.12^ab.A^	134.83 ± 6.96^a.A^	nd	362.93 ± 22.99^a.A^	119.78 ± 7.59^a.A^	17.43 ± 0.35^bc.A^
	P.os.	27.67 ± 1.20^a.A^	32.33 ± 0.88^ab.B^	279.73 ± 16.50^a.A^	105.36 ± 7.68^a.AB^	25.01 ± 3.45^bc.A^	410.09 ± 26.49^a.A^	135.34 ± 8.74^a.A^	5.51 ± 0.16^a.B^
	P.pl.	30.00 ± 0.58^a.A^	34.00 ± 1.00^a.B^	271.49 ± 11.94^a.A^	83.64 ± 6.30^a.B^	26.04 ± 3.68^ab.A^	381.16 ± 21.37^a.A^	125.80 ± 7.05^a.A^	3.41 ± 0.21^a.C^
	A.cl.	39.67 ± 0.88^ab.B^	25.67 ± 0.88^a.A^	280.03 ± 20.58^a.A^	97.87 ± 10.20^a.AB^	nd	377.91 ± 30.62^a.A^	124.72 ± 10.10^a.A^	2.36 ± 0.11^b.C^

cTPOMW 40%	P.er.	39.67 ± 0.88^ab.B^	26.67 ± 0.33^a.A^	266.96 ± 20.66^a.A^	96.73 ± 4.88^b.A^	nd	363.69 ± 24.71^a.A^	120.03 ± 8.16^a.A^	17.35 ± 0.86^bc.A^
	P.os.	30.00 ± 0.58^ab.A^	34.00 ± 0.58^ab.B^	245.06 ± 17.84^ab.A^	102.04 ± 4.27^a.A^	33.01 ± 3.58^ab.A^	380.10 ± 23.60^a.A^	125.45 ± 7.79^a.A^	5.45 ± 0.19^a.B^
	P.pl.	32.00 ± 0.58^ab.A^	35.00 ± 1.16^a.B^	255.60 ± 9.78^ab.A^	68.71 ± 7.19^ab.B^	28.72 ± 2.74^a.A^	353.03 ± 13.32^ab.A^	116.51 ± 4.39^ab.A^	3.83 ± 0.11^a.BC^
	A.cl.	40.67 ± 0.33^ab.B^	27.33 ± 1.20^ab.A^	274.36 ± 9.33^a.A^	78.05 ± 5.10^ab.AB^	nd	352.41 ± 11.89^a.A^	116.31 ± 3.92^a.A^	2.34 ± 0.08^b.C^

cTPOMW 60%	P.er.	43.00 ± 1.53^bc.B^	28.67 ± 0.78^ab.A^	199.66 ± 10.40^ab.A^	66.57 ± 5.70^bc.A^	nd	262.89 ± 15.32^ab.A^	86.76 ± 5.05^ab.A^	17.35 ± 0.86^bc.A^
	P.os.	34.67 ± 0.88^b.A^	36.33 ± 0.88^ab.BC^	192.17 ± 17.71^bc.A^	59.27 ± 9.41^b.A^	16.03 ± 1.84^cd.A^	267.47 ± 28.37^bc.A^	88.27 ± 9.36^bc.A^	5.92 ± 0.18^a.B^
	P.pl.	36.33 ± 1.20^b.A^	39.00 ± 0.58^a.C^	184.82 ± 16.55^b.A^	44.83 ± 4.90^bc.A^	16.57 ± 2.46^bc.A^	246.22 ± 22.72^c.A^	81.26 ± 7.50^c.A^	4.22 ± 0.06^a.BC^
	A.cl.	43.00 ± 0.58^b.B^	32.33 ± 1.45^bc.AB^	236.32 ± 15.00^ab.A^	54.82 ± 4.69^bcd.A^	nd	291.14 ± 19.69^ab.A^	96.09 ± 6.50^ab.A^	2.40 ± 0.15^b.C^
